# Arginine concentration in arterial vs venous blood in a bleomycin-induced lung inflammation model in mice

**DOI:** 10.1371/journal.pone.0285770

**Published:** 2023-05-12

**Authors:** Slobodan Tepic, Daniel Arens, Tim Buchholz, Dirk Nehrbass, Olivera Cvetkovic, Martin J. Stoddart, R. G. Richards, Stephan Zeiter

**Affiliations:** 1 Hepius Biotech AG, Zurich, Switzerland; 2 AO Research Institute, Davos, Switzerland; Ataturk University Faculty of Medicine, TURKEY

## Abstract

Pneumonia, always a major malady, became the main public health and economic disaster of historical proportions with the COVID-19 pandemic. This study was based on a premise that pathology of lung metabolism in inflammation may have features invariant to the nature of the underlying cause. Amino acid uptake by the lungs was measured from plasma samples collected pre-terminally from a carotid artery and vena cava in mice with bleomycin-induced lung inflammation (N = 10) and compared to controls treated with saline instillation (N = 6). In the control group, the difference in concentrations between the arterial and venous blood of the 19 amino acids measured reached the level of statistical significance only for arginine (-10.7%, p = 0.0372) and phenylalanine (+5.5%, p = 0.0266). In the bleomycin group, 11 amino acids had significantly lower concentrations in the arterial blood. Arginine concentration was decreased by 21.1% (p<0.0001) and only that of citrulline was significantly increased (by 20.1%, p = 0.0002). Global Arginine Bioavailability Ratio was decreased in arterial blood by 19.5% (p = 0.0305) in the saline group and by 30.4% (p<0.0001) in the bleomycin group. Production of nitric oxide (NO) and citrulline from arginine by the inducible nitric oxide synthase (iNOS) is greatly increased in the immune system’s response to lung injury. Deprived of arginine, the endothelial cells downstream may fail to provide enough NO to prevent the activation of thrombocytes. Thrombotic-related vascular dysfunction is a defining characteristic of pneumonia, including COVID-19. This experiment lends further support to arginine replacement as adjuvant therapy in pneumonia.

## Introduction

Even before the Covid-19 epidemic, infective pneumonia in the developed world was the most common cause of death due to infections, and the number four cause of death overall [1]. According to the CDC [2], there were 1’485’000 community-acquired pneumonia cases diagnosed in United States emergency departments in 2018, resulting in 47’872 deaths (3.2%). A prospective study of pneumonia cases hospitalized from June 1, 2014, to May 31, 2016, in all adult hospitals in the city of Louisville, Kentucky, reports a much higher pneumonia-related mortality [3]. Of the 7’449 patients enrolled in the study, 6.5% died while in hospital, 13.0% at 30 days, 23.4% at 6 months, and 30.6% at 1 year. A comprehensive review of literature on complications caused by thrombotic-related vascular disease suggests a causative relation to infectious pneumonia [1]. Pneumonia increases the risk of myocardial infarction, ischemic stroke, venous and arterial thrombosis, and pulmonary embolism, both in the acute phase of infection and later, with a significant increase in mortality post-hospitalization.

COVID-19 in the United States more than doubled the number of annual hospitalizations for pneumonia. From early 2020 until mid-2022, there were over 90 million confirmed cases, over 5 million hospitalizations, and over 1 million deaths [4]. The approximate, average number of annual hospitalizations due to COVID-19 was thus 2 million. The death rate was 20% relative to the number of hospitalized cases. Mortality due to COVID-19 when expressed as a percentage of seriously ill patients requiring hospitalization, or even intensive care, is in the range of other types of pneumonia. Complications are also very similar and closely correlated to thrombotic-related vascular disease [5]. Notwithstanding the particulars in clinical manifestations of different types of infective pneumonia and such obvious features of the causing pathogens as infectivity, it appears that there is an underlying commonality in lung inflammation deserving extra attention.

Molecular biology advancements of the last decades have produced large, detailed catalogs of intra and inter-cellular signaling networks in different classes of immune cell populations. The effector mechanism to attack pathogens directly as well as the host cells damaged by them relies on increased expression of inducible NOS (iNOS) and the rate of synthesis of its highly toxic product, NO [6]. The toxicity of NO depends on its concentration–at low concentrations, it is the most ubiquitous signaling molecule [7]. In addition to iNOS, two other enzymes produce NO but as a signaling molecule: endothelial NOS (eNOS) (also called constitutive, or cNOS) and neuronal NOS (nNOS). Endothelial NOS can synthesize NO into only a nanomolar range; iNOS into micromolar [8]. NO produced by endothelium suffices to prevent thrombocytes from activation and by its vasodilatory effect to balance the action of pressor peptides, all crucial for the stability of hemodynamics. It is important to consider the physical scales to appreciate just how critical the function of eNOS is. The surface area of the capillary bed in a human is about 1’000 m^2^ and it is all covered by endothelial cells producing NO. The half-life of NO in presence of erythrocytes is on the order of a millisecond [9]. The laminar flow within the capillary bed results in zero flow velocity along the capillary walls. The only reason cells of the size of thrombocytes move at all near the walls is that shear in the blood flow rolls them along–very slowly. The failure to locally produce NO would increase the risk of activation of thrombocytes, rolling by the endothelium with the NO deficit on a sub-millimeter scale. Activation is a misnomer–NO and prostacyclin, as extracellular signals, maintain active intracellular synthesis of cGMP and cAMP, respectively [10]. It is thus a constant activity of these two biochemical processes that prevent the “activation” cascade. This “activation” is terminal and can result in clot formation anywhere in the vascular system, as activated thrombocytes enter and are transported by the blood flow. Intracellular synthesis of NO by thrombocytes is insufficient to compensate for the lack of extracellular NO signal and would also be affected by the lowering of free arginine, the only substrate for NO synthesis [11]. The systemic defense against this fundamentally unstable clotting system is the constant surveillance of the vascular system by plasminogen/plasmin [12]. NOS enzymes convert arginine into NO and citrulline [13]. Arginase is one of the urea cycle enzymes (Arginase type I, found mostly in the liver). It converts arginine into ornithine and urea [14]. Other than its main systemic role of ammonia removal by the urea cycle, arginase (also as Arginase type II) plays other roles in many cells, including the modulation of NOS activity [15].

In the lungs, the capillary bed is formed by endothelial sheets covering 100 m^2^ of the epithelium of the air-exposed alveoli, rather than tubes found in all other organs [16]. The presence of pathogens, such as viruses, bacteria, or fungi triggers infiltration of this extremely thin, fragile tissue layer by immune cells. Endothelial and epithelial cells as well as infiltrated immune cells draw on free arginine in the blood plasma to produce NO. Excess NO in exhaled air in COVID-19 patients has been documented early during the pandemic [17]. Highly reactive NO in the presence of oxygen forms even more toxic NO_2_. The extremely short half-life of NO in presence of erythrocytes (due to NO reaction with hemoglobin), coupled with an oxygen-rich environment in the lungs, will further spatially restrict the effects of NO even if locally present in high excess. Should iNOS use enough free arginine it may well deprive eNOS of arginine. The predictable downstream effect would be thrombocyte activation with systemic effects.

The first report on general metabolites in COVID-19 patients was published in January 2021 by a French team in Rennes [18], followed a month later by a publication from Wuhan [19], China. The Rennes report showed a reduced concentration of arginine in 13 severely ill COVID-19 patients and 13 with moderate pneumonia compared to 13 healthy volunteers. Blood samples were collected at admission and on the 4^th^ and 7^th^ day of hospitalization. The largest reduction of arginine was at admission: 26% and 54% for moderate and severe groups respectively vs the healthy group. On day 4, the reductions were 11% and 40%, and 17% and 23% on day 7. T-cell number correlated with arginine concentration, i.e., it was lowered in COVID-19 patients. There was also a significant increase in plasma arginase activity at admission in the severe group (indirectly assessed by a ratio of ornithine to arginine). T-cell proliferative capacity measured *in vitro* from COVID-19 patients was significantly reduced and could be restored by arginine supplementation [18]. Based on these findings, the Rennes report authors raised the possibility of supplementation of arginine as adjuvant therapy for COVID-19 patients in intensive care.

The Wuhan report documented changes in metabolites from 45 blood samples collected from 28 patients hospitalized for COVID-19, 21 of whom returned for follow-up analysis after 30 days of recovery, compared to 48 healthy subjects [19]. Standard blood hematology and biochemistry parameters were compared to controls. Of the 18 amino acids measured, 14 had a significantly different concentrations in hospitalized patients. Only methionine was decreased, and 13 others were elevated. No data for arginine was presented.

The third report on amino acids in COVID-19 patients was published in June 2021 [20]. In a prospective study, two cohorts of SARS-CoV-2 positive patients hospitalized in Atlanta, Georgia were compared to 28 healthy controls. One cohort had 32 adults with COVID-19, the other (pediatric) had 20 children/adolescents, of which 9 had COVID-19, 9 had multisystem inflammatory syndrome (MIS-C), and 2 were asymptomatic but tested positive in admission screening. Arginine and citrulline in adult COVID-19 patients compared to controls were significantly lowered by 37 and 30%, respectively. In pediatric patients, these reductions were also statistically significant and even larger: 44 and 63%, respectively. In addition to arginine and citrulline, 9 other amino acids of the 21 measured, were significantly reduced in adult COVID-19 and 8 in the pediatric group. Two ratios considered relevant to immune response/competence were also significantly reduced: the arginine-to-ornithine ratio by 46 and 49% in adult and pediatric groups, respectively; and the global arginine bioavailability ratio, defined as GABR = c_arg_/(c_cit_+c_orn_), by 43 and 42% in adult and pediatric groups, respectively. The authors suggested, as did the ones of the Rennes study, that arginine adjuvant therapy should be considered, but have also stated that their study had not provided direct evidence for arginine depletion by the COVID-19 inflammation in the lungs rather than by some other, systemic disease processes.

This study of an experimental lung inflammation model in mice was undertaken in part to provide direct evidence that arginine depletion is due to a lung-centered process, if not to exclusion of other organs. The bleomycin model was chosen to avoid the complexities that come with handling experimental animals treated with infectious agents. The immune response thus triggered is only by the injury to the host cells and is likely of lower intensity than in lung infections. However, the model does have the main hallmarks of pneumonia in its local and systemic manifestations, [21], and hence is expected to contribute to the understanding of the pathology of pneumonia, which with COVID-19 has caused major medical, economic, and societal crises around the world.

## Materials and methods

There are several well-established experimental models for lung inflammation in mice. The bleomycin-induced lung inflammation was chosen to avoid the complexities of working with infectious agents. A recent publication [21] tracked mice’s body weight loss and body condition for approximately two weeks post-induction with bleomycin, a fibrogenic antibiotic, and saline. That model was successfully reproduced in this study, approved by the Ethics Committee for Animal Experimentation of the canton of Grisons (GR 07/2022).

### Experimental animals

Female mice C57BL/6J, older than 12 weeks from Charles River, Sulzfeld, Germany, were examined by animal care upon arrival at the AO Research Institute, their health certificate was checked, and they were allowed a minimum of two-week acclimatization period. Animals were not handled other than during cage changes by the animal caretaker. They were housed, three per cage, in individually ventilated cages (IVC, Tecniplast S.p.A., Buguggiate, Italy) with a 12-hour light/dark cycle and were fed *ad libitum* a maintenance diet from KLIBA NAFAG, Granovit AG (number 3436). Each cage had a water bottle that allows drinking *ad libitum*. The cages were checked daily by the caretakers to ensure all animals had enough water and food available. As an enrichment material, the mice got a plastic house and wood for gnawing on in their cages. They were marked using color markings on the tail applied during the instillation of saline or bleomycin. Cages were also marked correspondingly.

Six mice were randomly allocated to the saline (control) group and 12 to the bleomycin group. Food and water were never withheld from the animals.

### Bleomycin/saline instillation protocol

Bleomycin or saline application was done under deep sedation by using Medetomidine (0.5 mg/kg) and Midazolam (5 mg/kg) subcutaneously. The anesthetized animal was placed on an angled board (approximately 60° from the benchtop) and suspended by its front teeth. The tongue was moved out of the way by flat forceps, and the second pair of forceps was used to open the mouth. A 22-gauge intravenous catheter was introduced into the trachea. Once the catheter was positioned in the trachea, each mouse received a single instillation of 100 μL of saline with or without 0.075 U of bleomycin (1 U USA = 1000 IU). The mice were recovered in their home cages with their previous cage mates. Cages were placed in a warming cabinet (Aria Ventilated Cabinet, Tecniplast S.p.A., Buguggiate, Italy) and were observed continuously for 15 min and then every 30 min. The mice fully recovered within 1 hour and were afterward placed back in the IVC rack.

### Postoperative care

Housing and feeding remained the same as during acclimatization. Using a predefined score sheet (on general behavior, body temperature, respiration, appetite, and defecation), an animal caretaker or veterinarian scored the mice twice a day for the first 3 post-instillation days, followed by daily scoring. The animals were monitored for their general and eating behavior. The respiration, eyes, fur, and feces were also monitored. The body weight was measured daily after instillation. The results were noted on the score sheet. No animal reached the score that would call for early euthanasia.

### Blood sampling

Blood sampling was done under Sevoflurane anesthesia according to internal SOP number PESS 053/04 "Mouse and rat anesthesia" by induction with Sevoflurane in a Plexiglass box (ca. 7% in O_2_, flow rate 1 L/min), maintained with Sevoflurane through a face mask (ca. 2–3% in O_2_, flow rate 0.6–0.8 L/min). Analgesia was achieved by using Methadone (2.5mg/kg sc) plus Carprofen (5mg/kg sc). Under deep anesthesia, a carotid artery (left) and the abdominal (caudal) vena cava were surgically isolated. Blood collection was performed first from the carotid artery and then from the vena cava. A longitudinal incision was made in the blood vessel using a bent tip of a 23G needle. The arterial blood was collected immediately as it bled from the incision using a microtip on a pipette (PIPETMAN Classic P200, Gilson Inc.). The venous blood sample was taken by inserting a 30G needle attached to a 1 ml syringe into the vena cava. Venous blood was then drawn up very slowly to avoid occlusion of the vessel.

About 200 μL of each, arterial and venous blood were transferred into micro EDTA tubes pre-chilled in ice/water. Mice were euthanized via cervical dislocation and exsanguination. A macroscopic examination of the lungs verified inflammatory processes in all mice from the bleomycin group. Sample organs from each group were preserved for histopathology.

### Tissue processing and semiquantitative histopathological analysis

After macroscopic evaluation, tissue samples of lungs, heart, and brain, of both, saline- and bleomycin-treated animals (N = 2, each) were sampled for post-mortem microscopic evaluation. All samples were fixed in 4% buffered formaldehyde, dehydrated through an ethanol gradient, embedded in paraffin, sectioned to a thickness of 5 μm, and stained with H&E. All slides were analyzed in a blinded manner (without knowing the group) by a certified veterinary pathologist applying a semi-quantitative 6-scale grading system (grade 0–5) using a light microscope (BX40, Olympus, Tokyo, Japan) under brightfield-illumination focusing on the local tissue reactions with special regard to inflammatory as well as vaso-occlusive tissue reactions. Data compilation was performed directly during the histopathological examination, recording the findings on a Microsoft Excel worksheet. Representative images were taken using a microscope camera and image acquisition software (DP21 + cellSens, Olympus, Tokyo, Japan).

### Blood sample preparation and analysis

The blood was centrifuged at 5°C, 5000 g, for 10 min (Eppendorf, Centrifuge 5424 R, Eppendorf AG, Germany). Plasma was transferred into Eppendorf tubes, mixed with 10% sulfosalicylic acid (2 parts plasma + 1 part acid), left in a fridge for 30 minutes, and centrifuged again (5°C, 21130 g, 10 min). The supernatant was frozen and sent to Hepius Biotech AG, for amino acid analysis. The final volume of samples was 80 to 100 μL, which was too small for filtration. The samples were thawed out and left at 4–8°C overnight, before the final centrifuging (Eppendorf, MiniSpin, Eppendorf AG, Germany; 12100 g, 10 min). Supernatants were collected and transferred into micro tubes (Micro vial 1.1 ml, with Micro insert 0.1 ml, VWR, Germany) for the amino acid analyzer (LA8080 High Speed Amino Analyzer, Hitachi, Japan). A full chromatography run for each sample took 165 min. Data analysis was performed by OpenLAB 2 software (Agilent, Santa Clara, CA). Sample chromatograms were compared to a calibration standard (Pickering Laboratories, Mountain View, CA). The final selection included 19 amino acids and urea.

Statistical analysis was performed with the free, online software GraphPad, Dotmatics, (https://www.graphpad.com/quickcalcs/ttest1.cfm). All comparisons were checked for statistical significance with paired student t-tests within the groups, or unpaired student t-tests across the groups. The two-tailed p-value was calculated for all comparisons and is noted on all graphs and in the excel file in S1 File. Differences with a p-value <0.05 were considered significant. All concentrations were measured in blood plasma, but in the text, for brevity, reference to plasma is sometimes omitted and only arterial or venous blood is used to describe the samples.

## Results

### Body weight loss and blood sample analysis

**Fig 1:**
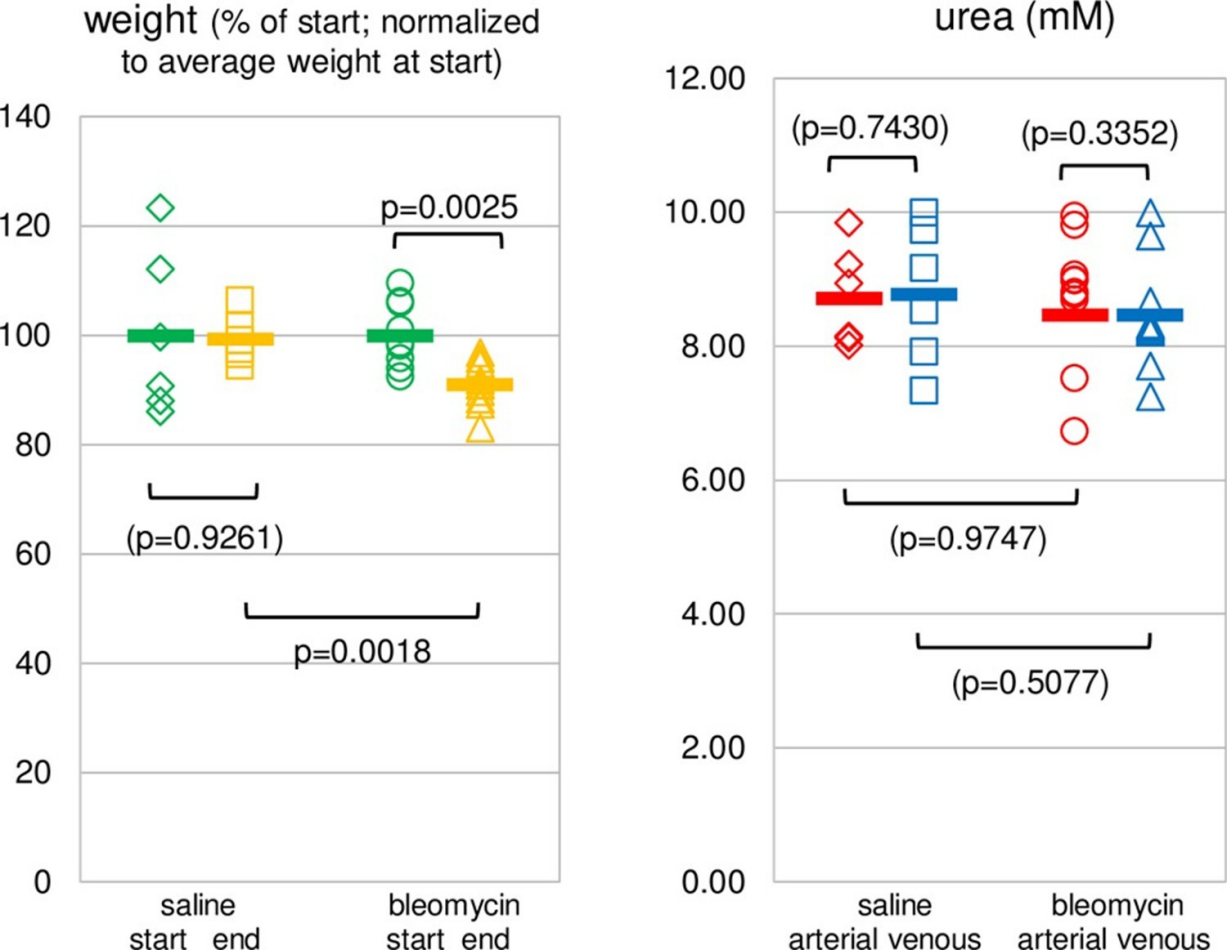
Weight loss from day 0 to day 6 and urea at day 6. (**A**) Body weight losses from start (day 0, green) to end (day 6, yellow) are shown for saline (6 animals) and bleomycin (10 animals) groups. The loss in the bleomycin group was 9% and significant. Values at the end, as well as at the start, are shown normalized to the averages at the start. (**B**) Urea concentrations in arterial (red) and venous (blue) blood samples for all mice in the saline and bleomycin groups. There were no significant differences in any comparisons.

The loss of body weight of the mice with bleomycin delivered to the lungs followed the expected trend and on day 6, before blood sample collection and euthanasia, it amounted on average to 9.0% (p = 0.0025) loss of the initial weight, Fig 1. The mice in the saline (control) group lost only 0.7% (p = 0.9261) of their initial weight. Of the 12 mice originally assigned to the bleomycin group, 2 were excluded due to technical issues in sample collection and processing. Concentrations of urea (in millimoles per liter, or mM) in the two groups are also shown in Fig 1. There was no statistically significant difference between any of the four sets of data (arterial and venous in the saline and bleomycin groups).

**Fig 2:**
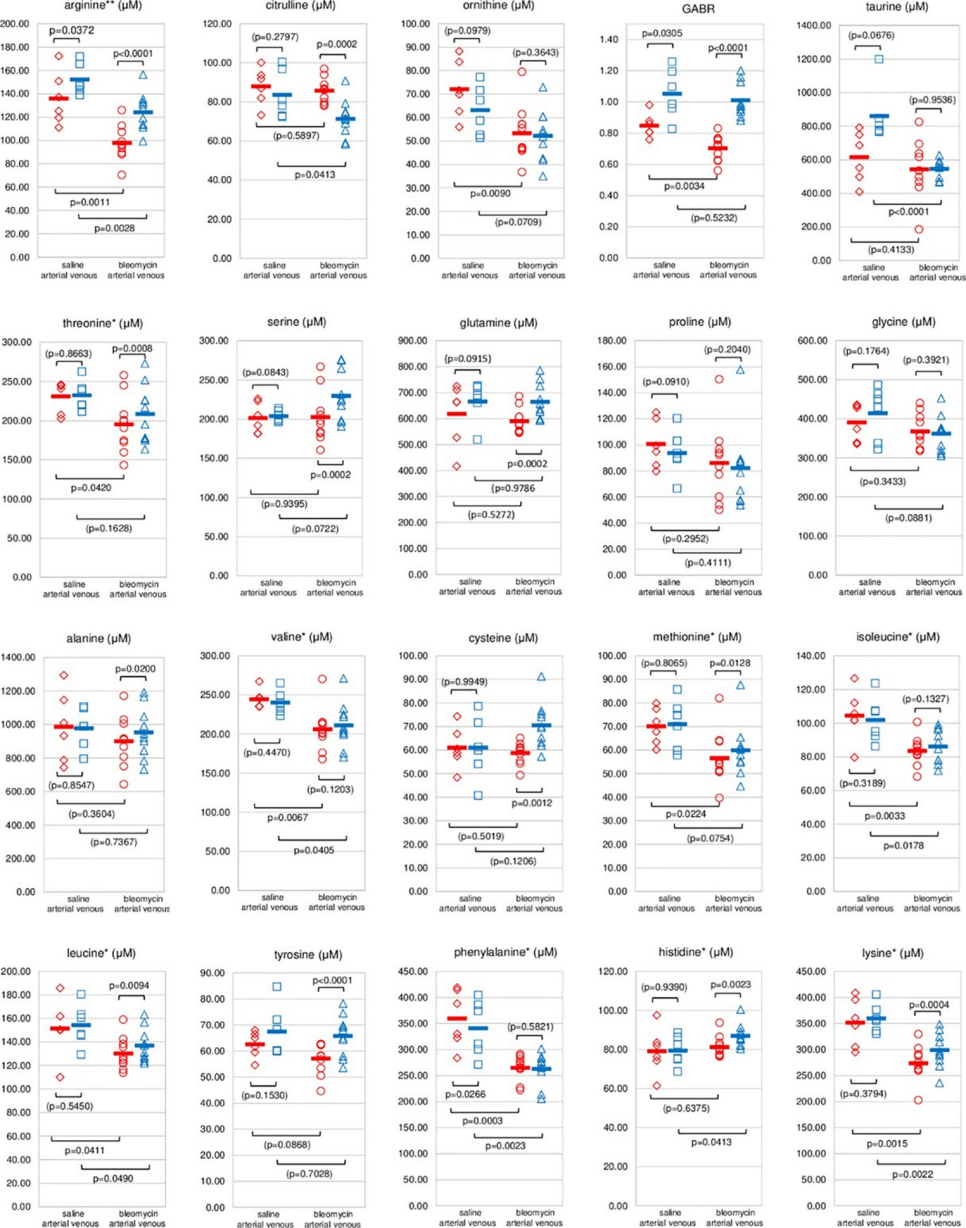
Concentrations of amino acids in arterial and venous blood of the saline and bleomycin groups. Values in the plasma (given as μmole/liter) of arterial blood sampled from the left carotid artery are shown with red symbols and the corresponding values for the venous blood from the caudal vena cava with blue symbols. The saline group with 6 animals is shown on the left side of the diagrams; the bleomycin group with 10 animals is on the right side. In addition to each individual value, the means of the groups are also plotted. P-values for student t-tests are written for each set of values compared. P<0.05 is considered statistically significant. Graphs show the data for 19 amino acids measured plus the calculated parameter GABR = c_arg_/(c_cit_+c_orn_). Essential amino acids are marked with*. Arginine, a semi-essential amino acid, is marked with **. P-values >0.05 are placed in parentheses.

Data from the analysis of 19 amino acids are shown in Fig 2 (in micromoles per liter, or μM). In the saline group, differences between venous and arterial concentrations reached statistical significance for only two amino acids. Arginine was lower in the arterial blood by 10.7% (p = 0.0372) and phenylalanine was higher by 5.5% (p = 0.0266).

In the bleomycin group, 12 of 19 amino acids analyzed had significantly different concentrations in arterial than in venous blood. Eleven of those were significantly lower. Only citrulline was higher (20.1%, p = 0.0002). Arginine was lower by 21.1%, p<0.0001. Other than differences between the arterial and venous blood concentrations, there were also differences, sometimes significant, between the groups, i.e., arterial blood in saline vs arterial in bleomycin. Arginine is the only amino acid that had statistically significant differences in all comparisons.

Arginine in the arterial blood in the bleomycin group compared to the arterial blood in the saline group was lower by 28.1%, p = 0.0011. For venous blood, this was 18.5%, p = 0.0028. Amino acids in the graphs in the first row, Fig 2, are of special interest in inflammation and blood clotting. As already mentioned, citrulline was significantly increased in arterial vs venous blood in the bleomycin group. It showed the same trend, but not significant, in the saline group. There was a significant reduction of citrulline in the venous blood of the bleomycin compared to the saline group by 14.6%, p = 0.0413. Ornithine was increased in the arterial blood of the saline group by 14.1%, but not significantly (p = 0.0979). In the bleomycin group, this increase was only 2.2%. However, there was a significant reduction of ornithine concentration in arterial blood in the bleomycin vs saline group by 25.9%, p = 0.0090.

The ratio of arginine concentration to the sum of citrulline and ornithine concentrations, the so-called Global Arginine Bioavailability Ratio, GABR, is considered an important parameter for the assessment of immunocompetence [22]. It is significantly reduced in both groups by passage through the lungs. In the saline group by 19.5%, p = 0.0305, and in the bleomycin group by 30.4%, p<0.0001. These reductions are obviously due to arginine concentrations being lowered and those of citrulline and ornithine increasing as the blood crosses the lungs.

Presumably, the citrulline increase is due to NOS and the ornithine increase is due to arginase activity. Comparing absolute molar changes, in the saline group arginine lost 16.3 μmole/liter, while citrulline and ornithine gained 4.3 and 8.9 μmole/liter, respectively. This leaves 3.1 μmole/liter of arginine to have been used in other reactions. In the bleomycin group, arginine lost 26.2 μmole/liter, while citrulline and ornithine gained 14.4 and 1.1 μmole/liter, respectively. This leaves 10.7 μmole/liter of arginine to be accounted for by other reactions. In summary, the production of citrulline in the lungs was increased in inflammation by 3.3-fold, and ornithine was decreased eightfold. The use of arginine for other reactions, such as for protein synthesis, was increased 3.5-fold.

Changes in other amino acids are of potentially high importance but are reported here for the benefit of future studies/analysis rather than as they might be related to blood clotting issues in pneumonia. The general observation is that the lungs are an organ of the perhaps unsuspected high level of amino acid metabolism, particularly in disease. An excel file with raw data of concentrations of all amino acids analyzed is available in S1 File.

### Histopathological analysis

**Fig 3:**
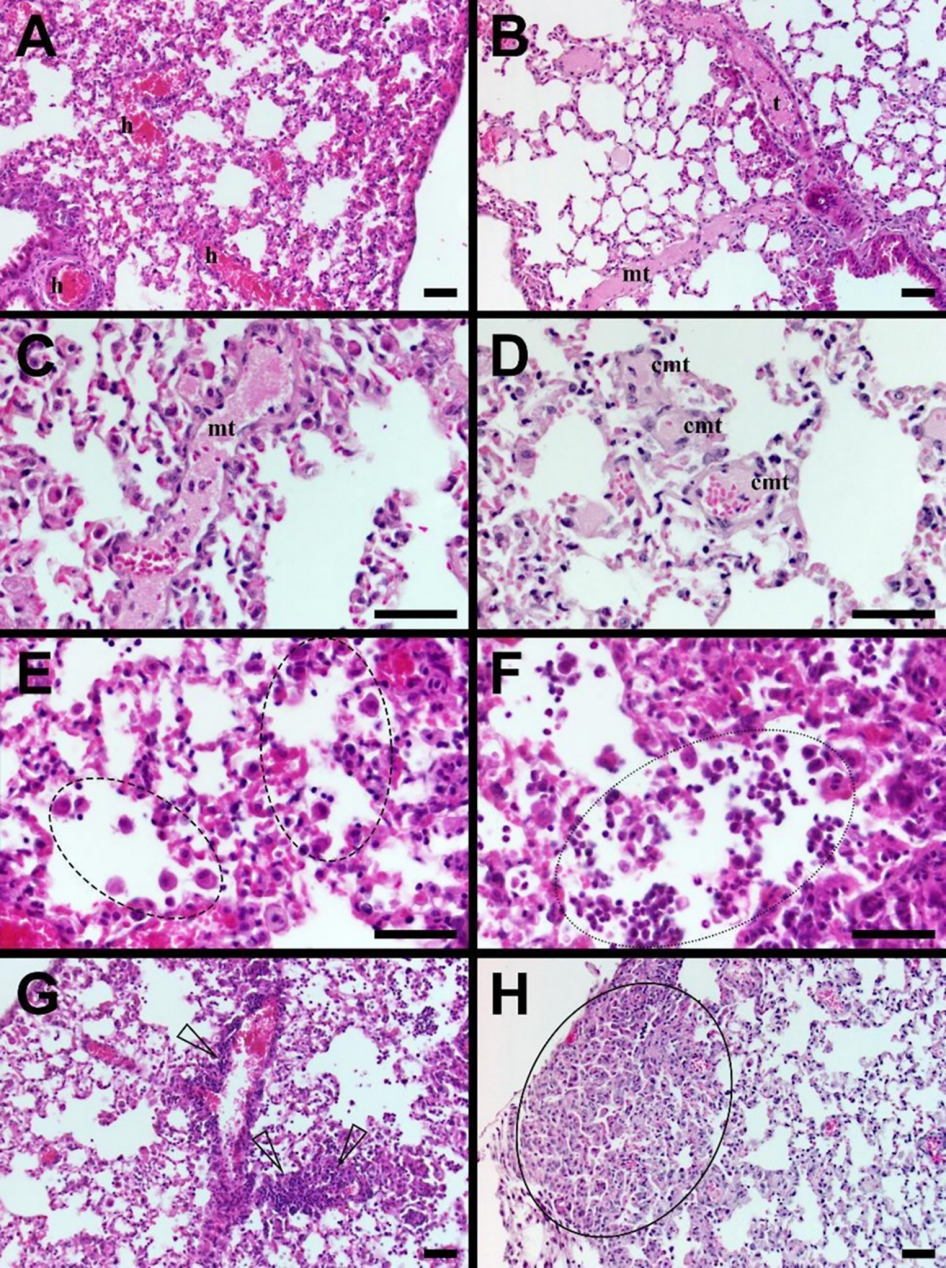
Histopathological changes related to the animal model used (bleomycin-induced model of lung inflammation at an early stage [day 6]). **(A)** increased vascular filling interpreted as hyperemia (**h**)**; (B-D)** vaso-occlusive changes: presence of venous thrombi (**t,** note the thin muscular layer of the venous wall), microthrombi (**mt**), and increased number of capillary microthrombi (**cmt**)**; (E)** increased alveolar macrophages (**dashed circle**)**; (F)** presence of alveolar granulocytes (**dotted circle**)**; (G)** increased peri-vascular lymphocytic infiltration (**arrow heads**)**; (H)** hyperplasia of pneumocytes type 2 (**circle**). Paraffin-embedded, HE-stained thin-sections, all scale bars 50 μm; objective 20x (A, B, G, H) and 50x (C, D, E, F).

The microscopic changes observed are all related to the animal model used (bleomycin-induced model of lung inflammation at an early stage (day 6)). They consisted in an increased vascular filling (interpreted as hyperemia) associated with vaso-occlusive changes (presence of venous thrombi and microthrombi, increased number of capillary microthrombi), Fig 3. These changes were accompanied by increased alveolar macrophages, presence of alveolar granulocytes, increased peri-bronchiolar/-vascular lymphocytic infiltration, hyperplasia of pneumocytes type 2, as well as by follicular hyperplasia of the mediastinal lymph node. An Excel worksheet with a semi-quantitative 6-scale grading system is available in S2 File.

## Discussion

This study was undertaken to provide experimental evidence for a hypothesis that metabolic processes of lung inflammation, common to different types of pneumonia, including COVID-19, can deplete the plasma level of arginine in the blood crossing the lungs and, in turn, possibly lead to clinically observed thrombotic complications. Well-characterized bleomycin-induced model of lung inflammation in mice was chosen for the *in vivo* experiment.

Plasma amino acids were measured in the blood samples collected from a carotid artery and vena cava from the same animal before euthanasia. Concentrations of 19 amino acids were compared between two groups of mice: the experimental group (10 animals) with bleomycin-induced lung inflammation and the control group (6 animals) with saline instilled into the lungs. Of the amino acids measured, only arginine concentrations significantly differed in all comparisons: from venous to arterial in the bleomycin and saline groups and between the groups, from venous to venous and arterial to arterial. The uptake level of arginine from the blood suggests its use over the need for protein synthesis. The compelling evidence for use of arginine by the immune response centered on the lungs is from the significantly increased arterial concentration of citrulline, the byproduct of nitric oxide synthesis by the immune effector cells. Ornithine, the product of arginine conversion to urea by arginase is also increased in arterial blood but the differences did not reach the level of significance with the number of animals in the groups. The GABR parameter calculated from all three of these amino acids that are directly involved in the process of the inflammatory response to lung injury showed a significant reduction, in the bleomycin group to the level considered insufficient for a fully functional immune system.

In the saline group, only two amino acids showed a significant change in concentration from venous to arterial blood–arginine was reduced by 10.7% and phenylalanine was increased by 5.5%. In the bleomycin group, concentrations of 12 amino acids significantly differed between venous and arterial blood– 11 were lowered and that of citrulline was increased by 20.1%. Arginine decreased the most, by 21.1%.

The significant reduction of arginine in the venous blood of the bleomycin compared to the saline group (-18.5%) may result from the failure of the systemic response to keep up with the demand for arginine centered on the lungs. Endogenous production of arginine via the intestinal-renal axis whereby citrulline is produced in the small intestine from glutamine or proline and converted to arginine by the kidneys, [23], is insufficient to meet the demand. This leads to a release of catabolic hormones from the liver and the breakdown of disposable proteins in muscle tissue, evidenced by body mass loss. This may still fall short of fully compensating for use of arginine and bring its global concentration significantly below the normal level.

Depletion of arginine may affect the course of inflammatory lung diseases in two major ways: by hindering the immune response and by causing activation of thrombocytes. Blood clots are universally found in autopsies of patients who have died of COVID-19, [24]. Some of them died from Disseminated Intravascular Coagulation (DIC), which should immediately raise an alert to arginine depletion. The blood clots, so-called microthrombi, are also found by advanced non-invasive techniques in many Long COVID patients examined by such means, [25]. Even though COVID-19 has greatly stimulated basic and applied medical research, it was mostly focused on the genetics of the pathogen(s) and the host. The role of basic metabolites in COVID-19 patients was initially ignored. The first findings were published only one year after the start of the pandemic and another year and a half later, only a handful of studies have addressed this important aspect. Whatever the process of depletion may be, it is a common, time-tested medical approach to such conditions to replenish what is missing.

The animal model chosen for this study is inadequate to fully account for the pathological processes in the lungs exposed to infective pathogens like bacteria or viruses. It was chosen to avoid the complexities that come with handling experimental animals treated with infectious agents. The bleomycin-induced damage reliably leads to inflammation and fibrosis of the lungs in mice. Body mass loss is well characterized, with about a half due to muscle wasting and a half due to fat reduction [21]. This was considered a crucial feature for the model selection, suggesting that inflammation leads to catabolic processes likely to be initiated by the reduction of plasma arginine but also possibly of other essential amino acids.

Collection of venous blood from the abdominal section of the vena cava was the only practical option in mice but it failed to collect the venous blood entering the lungs directly.

Sampling the vena cava at the abdominal level caused the blood from the vessel to empty from both the distal and proximal sections, hence collecting the mixture of blood from most of the splanchnic organs and the hindquarter of the body muscles. However, the venous blood entering the right atrium and being pumped into the lungs also collects all the lymphatic drainage and venous blood from the cranial half of the mouse, including the head and the heart. In a catabolic state, it is fair to assume that the venous blood entering the lungs would have higher amino acid concentrations from the protein breakdown than the blood from the vena cava. This would in turn show even higher changes in concentrations of amino acids in the blood crossing the lungs.

In a series of publications in the early nineties [26–28] a team from the Department of Surgery, University of Florida, published their findings from animal experiments and clinical observations in human patients on glutamine and alanine release by the lungs in health and disease. In rats, they could cannulate a carotid artery and right ventricle and thus obtain samples of blood just entering and exiting the lungs. In septic patients, the glutamine release rate could increase by an order of magnitude, but only temporarily. In patients in which sepsis progressed to pneumonia or severe adult respiratory distress syndrome, the lungs failed to provide glutamine. Arginine metabolism was not addressed in these studies.

As the modern tools for molecular analysis, specifically liquid chromatography/mass spectrometry, became generally available, ushering in a new approach to the studies of metabolism, the so-called Metabolomics, research teams from Scripps Institutes, La Jolla, CA, University of California, La Jolla, and Washington University, St Louis, MO, took on a task to quantify differences in molecular content of arterial vs. venous blood from 20 healthy individuals [29]. In the first stage of untargeted metabolite profiling, 8811 metabolic features were identified in the samples of blood taken from the radial artery and the brachial vein. The changes would thus be due mostly to the passage of blood through the capillaries of the forearm muscles. In the second phase of the study with the targeted analysis, 36 metabolites were quantified and compared between the arterial and venous blood. Six amino acids showed statistically significant differences. Arginine was not one of those. It was higher in the venous blood by 6%, i.e., there was a net release of arginine from the muscle tissue into the blood.

It is worth noting that the venous blood routinely sampled in the clinical setting from the brachial vein is closer in its molecular content to the arterial blood than to the venous blood from the vena cava. In a catabolic state, there would probably be a net release of most if not all protein amino acids from the muscle tissue. Under those conditions, the levels of amino acids in the blood collected from the brachial vein would significantly overestimate the concentrations of amino acids in the arterial blood.

Extending this approach of large-scale molecular analysis to all major organs, research teams from Princeton University, Princeton, NJ, University of Cambridge, Cambridge, UK, and the University of Pennsylvania, PA, performed quantitative analysis of inter-organ metabolite exchange in fasted pigs [30]. Concentrations of 597 known metabolites were measured in arterial and venous drainage blood of 11 organs in 5 pigs. Of those known metabolites, 256 had significant A/V differences. Amino acid differences were presented for the liver, viscera, head, legs, heart, kidneys, and skin, but not for the lungs.

Protective effects of “Functional Amino Acids” (a name coined to describe a class of amino acids with recently discovered regulatory effects on biological systems) on apoptosis, inflammatory response, and pulmonary fibrosis in mice challenged with lipopolysaccharide (LPS) were evaluated for arginine, glycine, and glutamine at the China Agricultural University, Beijing, in collaboration with Texas A&M University, College Station, TX [31]. Six groups of 7 mice per group were: no treatment (control), saline, LPS, arginine pre-treatment+LPS, glycine pre-treatment+LPS, and glutamine pre-treatment+LPS. Pre-treatment was performed by exposure to aerosolized compound once daily for 7 days. The LPS exposure of 30 minutes was followed 24 hours later by euthanasia. The evaluation of the lungs was done by histology, and immunostaining to quantify apoptosis and expression of inflammatory cytokines and chemokines, as well as the accumulation of neutrophils and macrophages in lung tissues. Pre-treatment with arginine or glycine effectively alleviated LPS-induced lung injury.

A comprehensive review of the epithelial dysfunction in lung diseases by Guoyao Wu, Texas A&M, and his collaborators from the China Agricultural University, Beijing, published as a chapter in the 2020 book *Amino Acids in Nutrition and Health*, Springer Nature Switzerland, G. Wu (ed.), [32], cover in great detail the current knowledge of molecular networks that maintain the integrity of the alveolar side of the lungs. Amino acid metabolism plays an equally critical role for epithelium as for endothelium, including the roles of NO in health and immune protection in lung diseases. The role of functional amino acids in the maintenance of the alveolar barrier is given full attention, including the experimentally demonstrated positive effects of dietary supplementation by arginine in various pathological conditions. Even in those early days of the COVID-19 pandemic, the authors raised the possibility of using dietary supplementation or i.v. infusion of functional amino acids, including arginine, to alleviate the consequences of this viral infection of the lungs.

A paper comparing amino acids in 60 patients with community-acquired (bacterial) pneumonia on the day of admission and day 7, after 6 to 8 days of treatment in the Sanyudo Hospital, Yonezawa, Japan, to 93 healthy controls was published in July 2021 [33]. At admission, the sum of concentrations of the 23 amino acids measured was lowered by 27% compared to controls. This was due to 12 of the amino acids being markedly decreased. On day 7, the sum was 29% higher than in controls. Arginine was 44% lower at admission and 32% higher on day 7. Citrulline was 38% lower at admission and 24% higher on day 7. Phenylalanine was increased on both, the admission day (by 60%) and day 7 (by 21%). Considering the central role that amino acids play in the immune response; the author suggested the use of immuno-nutrition tailored to the stages of pneumonia.

An interim report on a randomized, double-blind, placebo-controlled, parallel-group study comparing standard therapy for COVID-19 to the therapy supplemented with oral L-arginine was published in September 2021 [34]. The study was to enroll 290 patients with severe COVID-19, hospitalized in Ospedali dei Colli, Naples, Italy. The publication covered the first 101 patients. Of those, 11 were transferred to ICU before they could start participating in the study with the remaining 90 equally divided into the study and the placebo groups. The study group received an oral supplement of 1.66 g of L-arginine twice daily. The primary outcomes were time on respiratory support at 10 and 20 days and hospitalization time. At 10 days, in the arginine supplement arm, 71% of the patients were on reduced respiratory support vs 44% in the placebo group (p = 0.01). The average hospitalization time in the arginine arm of the study was 25 days vs. 46 days in the placebo group (p<0.0001). Of the 45 patients evaluated in each of the study arms, there were no deaths in the arginine group while 3 patients died in the placebo group.

A report on a similar study, from another hospital in Naples, with similarly positive outcomes was published in January 2022 [35]. Several ongoing studies on oral nutritional supplements, including arginine, for COVID-19 and Long COVID [36], are also reporting interim positive outcomes.

There is overwhelming evidence, both from the basic understanding of biology, and physiology and from clinical data, that dynamics of amino acids supply and utilization by the lungs in lung inflammations play a crucial role in the course of and potential common path to the endpoint of the disease. Observations of the local and systemic sequelae of pneumonia, including that in COVID-19, point to arginine for two main reasons: (i) it plays a central role in thrombocyte physiology via its conversion into NO, hence in blood clotting disturbances, and (ii) it is a substrate in multiple biochemical pathways utilized by the immune defense. Interorgan transport of amino acids has been studied in health (mostly in animal models but also in human volunteers) and disease.

In a tour de force study in 20 patients undergoing surgery for pancreatic cancer at the Maastricht University Medical Centre, concentrations of amino acids were measured in the venous blood from splanchnic organs (portal vein, hepatic vein, superior mesenteric vein, inferior mesenteric vein, splenic vein, and renal vein) and compared to blood from the radial artery [37]. Arginine concentrations were statistically significantly different (shown here as arterial minus venous in % of arterial) for portal drained viscera (-12.4%), liver (+33.1%), and small intestine (+27.1%). The data confirmed the role of the intestinal-renal axis as the main source of endogenous synthesis of arginine but the study, unfortunately, did not include the lungs.

In another study of relevance, concentrations of amino acids were measured in the arterial and venous blood across the brain (from the brachial artery to the jugular vein) in 8 healthy volunteers [38]. All of the 16 amino acids measured showed uptake by the brain, 12 of them with significant differences between concentrations in the venous and arterial blood. The average uptake was 8.3% (compared to arterial); with a minimum of 4.2% for taurine and a maximum of 13.2% for citrulline. Arginine was not measured. The same authors have previously measured amino acid uptake by the splanchnic bed, forearm muscle, leg, and kidney [39–42].

Many of the clinical reports referenced above fail to mention the details of blood collection, including the site of collection. There are significant differences in plasma amino acids even in healthy subjects from samples from the radial artery to the brachial vein [29]. In a catabolic state, these differences may be more significant.

Another problem with the clinical reports is their failure to provide details on parenteral nutrition which is probably routine in most cases of severe pneumonia, and it would include amino acids. The processing of blood plasma and the laboratory techniques used to measure concentrations of amino acids are not standardized and could cause differences between the studies. For example, in the papers referenced above, the baseline concentration of arginine in control groups varies by a factor of two. Reference intervals for plasma amino acids are rather wide, [43], but artifacts of sampling and analysis of the samples could also contribute to differences between the control groups.

There is a large discrepancy between the data on amino acids in COVID-19 patients from Wuhan, [19], and those from Rennes, [18], and Atlanta, [20]. In the Wuhan patients, levels of most amino acids were significantly higher than in controls, whereas, in patients from Rennes and Atlanta, they were significantly lower than in controls. From what is presented in these publications it is not possible to identify probable reasons for this major difference.

An important, if obvious question, is how much lower will arginine get as the arterial blood flows from the lungs into different organs? Unfortunately, the study of amino acids already referenced, [37], failed to measure arginine uptake by the brain. The brain tissue is relatively rich in arginase, [44], so it stands to reason that arginine use would probably exceed that of an average of other amino acids, i.e., the reduction could be by a further 10% or more. The brain, as stated, is at an elevated risk of vascular insult correlated to acute or recent pneumonia. The other organ with an increased risk of blood clotting is the heart. The heart muscle, unlike skeletal muscles, also contains a relatively high amount of arginase, [45], and this is likely to further lower the concentration of arginine in the arterial blood arriving from the lungs.

Liver damage, common in COVID-19 as diagnosed by elevated liver enzymes ALT, AST, ALP, and GGT in the blood, [46], also leads to the release of arginase type I, contributing to arginine depletion and compounding the risk of thrombotic complications.

While further animal experimental studies, as well as clinical observational studies, are needed and will be planned and executed, it is of utmost urgency to carry out clinical studies with adjuvant therapy by replacement of arginine for patients with pneumonia, including COVID-19. The Naples study and other similar studies have used oral arginine replacement as the simplest, least demanding mode of intervention, but the outcomes could still be much improved with continuous i.v. infusion of e.g., arginine hydrochloride. Arginine hydrochloride for i.v. delivery is a registered product of at least two pharmaceutical companies but not for these indications. Another option to consider is the inhalation of arginine hydrochloride, which has been successfully, experimentally used in patients with cystic fibrosis, [47]. Arginine hydrochloride has not been registered for use by inhalation for any medical condition. The doctors responsible for treating COVID-19 patients are simply not in a position to use any drugs not specified by NIH guidelines which strongly discourages the use of any drug off-label, [48]. The most current issue of the guidelines of August 2022 does not even mention arginine. A clinical trial to extend indications of arginine hydrochloride to pneumonia, including COVID-19, deserves an urgent initiative.

## Supporting information

S1 File (XLSX)

S2 File (XLSX)
